# Body mass index does not affect response of rituximab in patients with rheumatoid arthritis: results from the TURKBİO registry

**DOI:** 10.55730/1300-0144.5698

**Published:** 2023-06-21

**Authors:** Ahmet KARATAŞ, Rabia PİŞKİN SAĞIR, Süleyman Serdar KOCA, Ediz DALKILIÇ, Gerçek CAN, Yavuz PEHLİVAN, Ayten YAZICI, Nevsun İNANÇ, Ayşe CEFLE, Zeynep ERTÜRK, Servet AKAR, Soner ŞENEL, Merih BİRLİK, Nurullah AKKOÇ, Fatoş ÖNEN

**Affiliations:** 1 Division of Rheumatology, Department of Internal Medicine, Faculty of Medicine, Fırat University, Elazığ, Turkiye; 2 Division of Rheumatology, Department of Internal Medicine, Faculty of Medicine, Uludağ University, Bursa, Turkiye; 3 Division of Rheumatology, Department of Internal Medicine, Faculty of Medicine, Dokuz Eylül University, İzmir, Turkiye; 4 Division of Rheumatology, Department of Internal Medicine, Faculty of Medicine, Kocaeli University, İzmit, Turkiye; 5 Division of Rheumatology, Department of Internal Medicine, Faculty of Medicine, Marmara University, İstanbul, Turkiye; 6 Division of Rheumatology, Department of Internal Medicine, Faculty of Medicine, Medipol University, İstanbul, Turkiye; 7 Division of Rheumatology, Department of Internal Medicine, Faculty of Medicine, Katip Çelebi University, İzmir, Turkiye; 8 Division of Rheumatology, Department of Internal Medicine, Faculty of Medicine, Erciyes University, Kayseri, Turkiye; 9 Division of Rheumatology, Department of Internal Medicine, Faculty of Medicine, Celal Bayar University, Manisa, Turkiye

**Keywords:** Body mass index, drug survival, obesity, rheumatoid arthritis, rituximab

## Abstract

**Background/aim:**

Adipose tissue produces several inflammatory mediators. Thus, obesity affects the disease course and the responses to the antirheumatic agents in inflammatory diseases. The aim of the study was to determine whether the body mass index (BMI) is involved in the response to rituximab in rheumatoid arthritis (RA).

**Materials and methods:**

This multicenter retrospective study included 206 RA patients who received rituximab from the Turkish Biologic (TURKBIO) registry between 2011 and the end of May 2017. Demographic and clinical data including age, sex, disease type, disease duration, and previous or current treatment with disease-modifying antirheumatic drugs (DMARDs) and biological drug durations are stored in the database. Patients with a BMI ≥30 kg/m^2^ were classified as obese, and patients with a BMI <30 kg/m^2^ were classified as nonobese. Kaplan-Meier survival analysis was performed to estimate the drug survival. The subgroups were compared using the log-rank test.

**Results:**

The mean BMI of 206 patients included in the study was 27.05 (17.2–43.4) kg/m^2^. There were 59 (28.6%) patients in the obese group and 147 (71.4%) patients in the nonobese group. The mean age, female percentage, and baseline disease activity score 28 (DAS28) were higher in the obese group than in the nonobese group. However, the ΔDAS28 at both 6 and 12 months were not significantly different between the groups (p = 0.785 and p = 0.512, respectively). Patient pain Visual Analogue Scale (VAS), patient fatigue VAS, and patient global VAS scores were also significantly higher at baseline in the obese group (p = 0.003, p = 0.006, and p = 0.006, respectively). However, no significant difference was found in terms of changes in patient pain VAS, patient fatigue VAS, patient global VAS and physician global VAS scores at 6 and 12 months compared to those at baseline. Rituximab treatment was ongoing for 71.2% of the obese and 63.3% of the nonobese patients (p = 0.279). The median drug survival duration was 77 months in the obese group and 62 months in the nonobese group (p = 0.053). The estimated drug survival rates for rituximab were not statistically significantly different in the obese and nonobese groups. Rituximab-related side effects were also similar between the groups.

**Conclusion:**

In obese and nonobese patients with RA, rituximab treatment exhibits similar side effects and similar long-term efficacy. These results suggest that obesity does not alter drug survival for rituximab and response rates, in RA and rituximab may be a favorable treatment agent in patients with RA and obesity.

## 1. Introduction

Obesity, which plays a role in many chronic systemic diseases and sudden cardiac death in young individuals, is a serious disease for the community [1, 2]. Research on obesity has increased over the last few years due to the increased prevalence of obesity and increased risk of obesity-related illnesses [3]. It is understood that white adipose tissue has been associated with systemic inflammation in the past 10 years [4,5]. In addition, white fat tissue behaves like an endocrine active organ. Adipose tissue is involved not only in metabolism but also in the immune system and inflammatory processes through proinflammatory mediators and adipocytokines, such as tumor necrosis factor (TNF)-α, interleukin (IL)-6, adiponectin, leptin, resistin, visfatin, and C-reactive protein (CRP) [6].

Rheumatoid arthritis (RA) is a chronic systemic inflammatory disease [7] and obesity is seen in 18%–31% of patients with RA and slightly higher than the general population. Almost 60% of patients with RA appear to be overweight [8, 9]. The relationship of obesity on the presence of RA and disease activity is still unclear [10,11]. In the early stages of RA, obesity may slow the progression of the disease [12]. However, obesity can increase disease activity and disability in patients with advanced RA [13]. In a large retrospective case-control study, obesity was found to cause a modest increase in risk for RA development in patients [14]. Additionally, studies in different patient cohorts have shown that obesity is associated with seronegative RA in females [15].

Considering the fact that RA is a progressive disease, the accumulation of joint damage can cause irreversible disability if left untreated or even improperly treated. Numerous RA patients all over the world are treated with conventional agents, such as glucocorticoids and methotrexate (MTX) [16]. Nonetheless, some patients may not tolerate them or even may not achieve disease remission. Although the etiology and the pathogenesis of RA are complex, different newly employed biologics have revolutionized therapeutic approaches. They usually suppress the immune system by targeting particular signaling pathways and act in a more specific manner. It was suggested that biologic agents, such as TNFα inhibitors and rituximab could significantly reduce mortality risk in RA patients compared to conventional synthetic disease-modifying antirheumatic drugs (csDMARDs) [17]. Rituximab, a chimeric anti-CD20 monoclonal antibody, depletes B cells through different mechanisms, including the apoptosis of B cells, complement-dependent cytotoxicity, and mediation of antibody-dependent cellular cytotoxicity [18]. Generally, rituximab administration in RA patients has been accepted as a safe procedure, and prolonged exposure to rituximab does not seem to be related to additional adverse events. However, there are some increasing concerns related to the safety profiles of rituximab in RA patients, such as the development/reactivation of infections, failure of immunization, and paradoxical reactions [19].

Patients with a body mass index (BMI) >30 kg/m^2^ are considered as obese. Pharmacokinetic variables such as drug clearance and distribution volume can be affected by obesity [20]. Several previous reports have documented that obesity decreases the response rate to anti-TNF-α agents in RA [21, 22]. Therefore, it is thought that adipose tissue has effects in the treatment of biological agents, and obesity affects the course of the disease and the response to antirheumatic agents in RA [21, 22].

Since previous studies have documented that obesity reduces the response rate to anti-TNF-α agents in RA, the aim in this study was to determine whether BMI affects the response to rituximab in RA.

## 2. Materials and methods

The Turkish Biologic (TURKBIO) database is the Turkish version of the Danish DANBIO rheumatology database established in 2011. This database collects demographic and clinical data such as age, sex, disease type, disease duration, disease activity, previous or current treatment and biological drug use duration from patients with RA receiving biological treatment at several tertiary rheumatology centers across the country. Ethics committee approval was obtained for this database and the study from the Turkish Medicines and Medical Devices Agency of the Ministry of Health dated 25/07/2013 under number 26247029-514-05-01. This multicenter retrospective study included 206 patients enrolled in the TURKBIO database who met the American College of Rheumatology/European League Against Rheumatism 2010 Rheumatoid Arthritis Classification criteria [23], receiving standard doses of 1000 mg rituximab on days 0 and 15 between 2011 and the end of May 2017.

Demographic data prior to rituximab treatment, disease duration, BMIs (the BMIs of the patients at the time of registration in the TURKBIO database), medications received before rituximab treatment, disease activity indices at baseline, and 6 and 12 months of rituximab treatment [disease activity score 28 (DAS28)], Health Assessment Questionnaire (HAQ) scores, Visual Analogue Scale (VAS) scores (including the patient pain VAS, patient fatigue VAS, patient global VAS, and physician global VAS scores), erythrocyte sedimentation rates (ESR), CRP level, and whether the patients were continuing rituximab treatment were recorded. Patients with a BMI ≥30 kg/m^2^ were classified as obese, and patients with a BMI <30 kg/m^2^ were classified as nonobese [2].

## 3. Statistics

Predictive Analytics for Windows 18.0 (Chicago, IL, USA) was used for the statistical analysis. The continuous variables were presented as either the mean ± SD or median (min–max or IQR) according to data distribution normality and the categorical variables were presented as numbers (%). Kaplan-Meier survival analysis was used for the descriptive statistics to determine the drug use rates, and the log-rank test was used to determine the difference between thee BMI groups. The chi squared test was used for the categorical variables, and Fisher exact test was used where the chi squared test was not applicable. The Mann-Whitney U test was used for the numerical variables when the data were not normally distributed. Cases in which the type-1 error level was <5% were interpreted as statistically significant.

## 4. Results

### 4.1. Demographic and baseline characteristics

There were 157 (76.2%) female and 49 (23.8%) male patients, and the mean age was 58 (21–85) years. The mean disease duration was 12 (2–36) years. Hypertension was the most prevalent comorbidity (33.6%), followed by osteoporosis, diabetes, depression, asthma, cardiovascular diseases, kidney diseases, chronic bronchitis, liver diseases, tuberculosis, and cancer. The mean BMI of the3 206 patients included in the study was 27.05 (17.2–43.4) kg/m^2^. There were 59 (28.6%) patients in the obese group and 147 (71.4%) patients in the nonobese group. The smoking history of 162 patients included in the study was known, 92 patients (56.8%) had never smoked, 36 patients (22.2%) had quit, and 34 patients (21%) were still smoking.

Rheumatoid factor and anticyclic citrullinated peptide antibodies were positive in 81.4% and 75.2% patients, respectively, and 158 (76.6%) patients were receiving combination treatment with rituximab. Of these patients, 91 (44.2%) were using MTX and 67 (32.5%) were using leflunomide combination treatments. When the biological naivety was evaluated, it was revealed that 152 patients had previous exposures to at least 1 biological agent, and the most commonly used biological agent was etanercept, at a rate of 25.7% (53 patients). There was no difference in the use of concomitant csDMARDs or previous biological treatments between the obese and nonobese groups.

The mean duration of rituximab treatment was 37 (2–150) months. While 135 patients (65.5%) continued treatment, 71 (34.5%) had discontinued. The lack of effectiveness was the most frequently reported reason for discontinuing treatment, and treatment was discontinued in 24 patients for this reason. Only 10 of the treated patients experienced side effects, of whom 4 had to discontinue treatment due to major side effects. The reasons for the patients to discontinue using the rituximab are shown in Table 1.

The median ESR was 32 (1–100) mm/h, CRP was 9 (0–87) mg/dL, total DAS28 was 4.45 (1.7–7.1), HAQ score was 1.2 (0–2.88), patient pain VAS score was 60 (0–100), patient fatigue VAS score was 50.5 (0–100), patient global VAS score was 60 (0–100) and physician global VAS score was 50 (0–90).

Baseline demographic, clinical, and laboratory data according to the BMI status of the patients are presented in the Table 2. The mean age and percentage of females were higher in the obese group. The difference in the smoking status between the BMI groups was statistically significant. The proportion of patients still smoking was low in the obese group (p = 0.007). Investigation of the baseline disease activity indicators showed that the DAS28 and patient pain VAS, patient fatigue VAS and patient global VAS scores were significantly higher in the obese group (p = 0.02, p = 0.003, p = 0.006, and p = 0.006, respectively). No difference was found in terms of the other parameters (Table 2).

### 4.2. Changes in disease activity with rituximab treatment

The values of the patients at baseline and 6 and 12 months, as well as the Δchange values at 6 and 12 months are presented in the Table 3. The DAS28 score was significantly higher in the obese group at baseline and 6 months (p = 0.02, p = 0.04, respectively). However, the ΔDAS28 scores at both 6 and 12 months were not significantly different between the groups (p = 0.785 and p = 0.512, respectively). The patient pain VAS, patient fatigue VAS, and patient global VAS scores were also significantly higher at baseline in the obese group (p = 0.003, p = 0.006, and p = 0.006, respectively). However, no significant difference was found in terms of changes in the patient pain VAS, patient fatigue VAS, patient global VAS, and physician global VAS scores at 6 and 12 months compared to those at baseline (p = 0.431, p = 0.971, p = 0.51, and p = 0.625 for changes in the scores between baseline and 6 months; p = 0.838, p = 0.463, p = 0.984, and p = 0.355 for changes in the scores between baseline and 12 months). Patients with obesity treated with rituximab had a greater decrease in delta CRP levels between baseline and 6 months than patients without obesity (p = 0.031).

### 4.3. Treatment retention rates

The mean duration of rituximab use was 39 (2–111) months in the obese group and 36 (2–150) months in the nonobese group (p = 0.105). In addition, 71.2% of the obese patients and 63.3% of the nonobese patients continued treatment (p = 0.279). Looking at the percentage of patients who continued treatment at year 1 and year 5, 100% of the obese patients and 99.5% of the nonobese patients continued treatment in year 1. However, the 5-year evaluation showed that 67.5% of the obese group and 52.8% of the nonobese group continued treatment. Lack of effectiveness was the main reason for discontinuing treatment in both groups, and there was no significant difference between the groups. Rituximab-related side effects were also similar between the groups. The median duration of drug use was higher in the obese group (77 months) than in the nonobese group (62 months) (p = 0.053); however, this difference was not statistically significant (Figure).

## 5. Discussion

The results of this study showed that only 32.5% of the patients were of normal weight (BMI <25) and 28.6% were obese (BMI ≥30), and the burden of disease was higher in the patients with obesity. Furthermore, the findings revealed that drug survival and response rates for rituximab in RA treatment do not change in the presence of obesity.

Giving the appropriate treatment to the appropriate patient is important to provide cost-effective care and prevent morbidity and mortality of the disease. The patient’s response to treatment is affected by numerous factors, and the impact of obesity, which is a serious health problem, on both disease occurrence and treatment response have been investigated in studies. The results from 2 large prospective cohort studies showed an increased risk for developing both seropositive and seronegative RA in patients with obesity. Interestingly, the duration of obesity was also found to be associated with this risk [24]. In the current study, only 67 (32.5%) patients with RA had a BMI <25 kg/m^2^ and were not obese or overweight. Since obesity is the cause of RA, it may be commonly observed in patients with RA. However, to the best of our knowledge, there are no studies in the literature showing that RA causes obesity, and corticosteroid treatment as well as the restriction of physical activities due to active arthritis may also be the reason for the more frequent incidence of obesity in patients with RA. With the understanding that obesity increases the risk of developing RA, whether it affects treatment response has also been investigated. Studies have shown that in addition to being a common comorbid condition, obesity causes the disease to worsen and also affects the treatment response to several agents [25]. Higher DAS28, patient pain VAS, patient fatigue VAS, and patient global VAS scores, which are among the pretreatment disease activity indicators, found in the obese group in the current study were in line with the literature. In a study by Colmegna et al. involving 200 patients with RA, the HAQ, DAS-28, and tender joint scores were higher in the obese group compared to those in the nonobese group [26]. Similarly, a study by Sandberg et al. that evaluated whether the presence of obesity at the time of diagnosis affects disease activity in the early stage of RA found higher DAS scores in patients with obesity [25]. In addition to the biological change caused by obesity, in the study by Sommers et al. [27], they observed that the pain threshold of patients with RA and obesity was lower, and the patients felt more desperate in the presence of pain. This may lead to higher DAS scores due to the low self-confidence of the patients in both managing arthritis and losing weight [27].

Since proinflammatory cytokines, including TNF and IL-6, are secreted by white adipose tissue, it is thought that excess adipose mass in patients with RA and obesity may affect treatment response by increasing the level of these cytokines [28]. Indeed, in a study by Schäfer et al. on patients with RA that investigated the relationship between obesity and response to conventional synthetic and biological DMARDs, it was seen that obesity reduced the efficacy of cytokine-targeted treatments such as TNF inhibitor and tocilizumab treatments, whereas such decreased efficacy was not observed for therapeutic agents targeting immune cells, such as rituximab and abatacept treatments [29]. However, in another retrospective study conducted by Pers et al., treatment responses after 6 months of tocilizumab use were not affected by the BMI [30]. A French study including 115 patients with RA who received tocilizumab found that treatment responses were not different between the BMI groups [31]. On the other hand, it was shown in an experimental study by Crouch et al. that obesity inhibits B cell development [32]. Both increased cytokine secretion and decreased B cell development in obesity may be the reason for the lack of effectiveness of rituximab treatment.

A study on 19,372 patients with immune-mediated inflammatory diseases, including inflammatory bowel disease (IBD), RA, spondyloarthropathies, psoriasis, and psoriatic arthritis, examined whether obesity affects response to anti-TNF agents. In that study, the treatment failure rate was 60% higher in the patients with obesity, but while this difference was seen in the patients with rheumatic diseases, it was not seen in those with IBD [33]. Again, in a study evaluating the efficacy of TNF inhibitor in the obese group among patients with ankylosing spondylitis, RA and psoriatic arthritis patients, a negative correlation was found between the BMI and response to anti-TNF treatments [22, 34, 35]. However, to date, the effect of anti-TNF agents on adipokine levels remains unclear. It is known that the pharmacokinetics of infliximab (IFX), an intravenously administered nonlipophilic anti-TNF molecule, may change in the presence of excess adipose tissue. Therefore, it can be thought that the distribution volume of a nonlipophilic drug such as IFX may be reduced due to the presence of excess adipose tissue, and this may partially explain its limited effect in patients with obesity compared to the effect of other subcutaneously administered anti-TNF agents (etanercept and adalimumab). However, in the same study, although the number of patients was limited, a high BMI did not show any adverse effects on treatment and clinical responses in 114 patients with RA receiving rituximab. Although rituximab is a nonlipophilic drug similar to IFX, the increase in adipose tissue had no impact on drug pharmacokinetics and rituximab distribution [36]. Although there are various studies showing that the efficacy of anti-TNF agents decreases in patients with obesity, no adverse outcomes were reported with rituximab treatment, similar to the presents study.

Since this study was retrospective registry data, it is not easy to find and present all the data of the patients, but it is important that this study was based on real-life data. However, the lack of power analysis was a limitation of the study, and all of the patients enrolled in a certain time interval were included in the study. In addition, another limitation of the study was that the patients had BMIs recorded at the time they were registered in the TURKBIO database, and their BMIs at the beginning or end of rituximab treatment were not known.

In conclusion, obesity is common in patients with RA, and obesity leads to a greater disease burden. In obese and nonobese patients with RA, rituximab treatment exhibits similar side effects and similar long-term efficacy. These results suggest that the long-term efficacy of rituximab treatment is not affected by obesity in patients with RA, and rituximab may be a favorable treatment agent in patients with RA and obesity. Nonetheless, these data should be further investigated in prospective case-control studies.

## Figures and Tables

**Figure:**
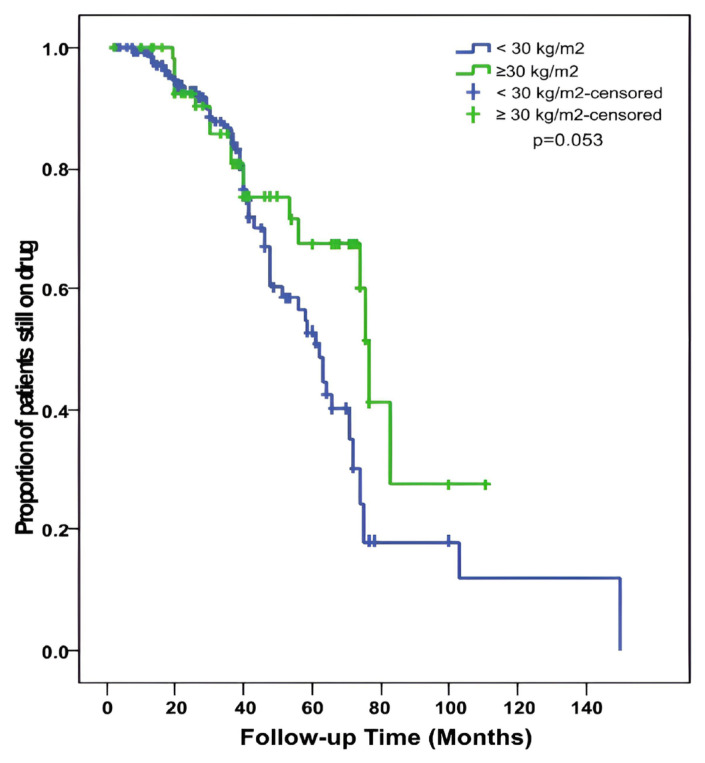
Drug survival curve in the nonobese (BMI <30 kg/m^2^, n: 147) and obese (BMI ≥30 kg/m^2^, n:59) patients.

**Table 1:** Reasons for discontinuing the rituximab.

	Obese patients (n = 17/59)	Nonobese patients (n = 54/147)	p-value (chi squared)
Lack of effectiveness (n, %)	8 (47.1)	16 (29.6)	0.325
Remission (n, %)	5 (29.1)	18 (33.3)	
Side effects (n, %)	2 (11.8)	2 (3.7)	
Being out of control (n, %)	1 (5.9)	3 (5.5)	
Infections (n, %)	1 (5.9)	2 (3.7)	
Surgical intervention (n, %)	0	1 (1.8)	
Others (n, %)	0	12 (22.2)	

**Table 2:** Baseline demographic, clinical, and laboratory data of the obese and nonobese patients.

	Obese patients (BMI ≥30 kg/m^2^) (n = 59)	Nonobese patients (BMI <30 kg/m^2^) (n = 147)	p-value
Age (years)	60 (34–80)	56 (21–85)	0.013
Disease duration (years)	10.5 (2–36)	12 (2–36)	0.713
Females (n, %)	53 (89.8)	104 (70.7)	0.004
Still a smoker (n, %)	6 (12.2)	28 (24.8)	0.007
RF positivity (%)	87.2	78.9	0.220
Anti-CCP positivity (%)	73.9	75.7	0.814
Basal ESR (mm/h)	34 (13–84)	32 (1–100)	0.199
Basal CRP (mg/dL)	10.5 (2–75)	8.5 (0–87)	0.383
Basal DAS28	4.8 (2–7.1)	4.3 (1.7–6.6)	0.020
Basal HAQ	1.15 (0.25–2.88)	1.25 (0–2.88)	0.869
Basal VAS - Patient pain	75 (40–100)	52 (0–100)	0.003
Basal VAS - Patient fatigue	68 (0–90)	50 (0–100)	0.006
Basal VAS - Patient global	72 (21–100)	50 (0–100)	0.006
Basal VAS - Physician	61.5 (6–90)	50 (0–90)	0.121

Data are expressed as the median (min–max). RF: rheumatoid factor, CCP: cyclic citrullinated peptide, ESR: erythrocyte sedimentation rate, CRP: C-reactive protein, DAS: disease activity score, HAQ: Health Assessment Questionnaire, VAS: visual analog scale.

**Table 3:** Comparison of the disease activity indices at baseline, 6 months and 12 months.

	Obese patients (n = 59)	Nonobese patients (n = 147)	p-value
ESR at baseline (mm/h)	34 (13–84)	32 (1–100)	0.199
ESR at 6 months (mm/h)	28 (5–73)	27 (2–110)	0.913
ESR at 12 months (mm/h)	25 (8–50)	28 (2–94)	0.980
**Δ**_1_ESR (mm/h)	−17.5 (−46 to 11)	−4 (−45 to 29)	0.114
**Δ**_2_ESR (mm/h)	−2 (−48 to 11)	−4 (−40 to 32)	0.686
CRP at baseline (mg/dL)	10.5 (2–75)	8.5 (0–87)	0.383
CRP at 6 months (mg/dL)	6 (2–70)	6 (0–105)	0.673
CRP at 12 months (mg/dL)	4.14 (3–27)	4 (0.9–46.3)	0.903
**Δ**_1_CRP (mg/dL)	−10 (−67 to 4)	0 (−69 to 67)	0.031
**Δ**_2_CRP (mg/dL)	−3 (−38 to 19)	0 (−54.7 to 28.3)	0.354
DAS28 at baseline	4.8 (2–7.1)	4.3 (1.7–6.6)	0.02
DAS28 at 6 months	3.4 (1.7–5.6)	2.7 (1.2–5.8)	0.045
DAS28 at 12 months	3 (1.–5.8)	2.5 (1.2–5.8)	0.274
**Δ**_1_DAS28	−1.6 (−4.4 to 0.4)	−0.9 (−4 to 0)	0.785
**Δ**_2_DAS28	−1.95 (−5.4 to 0.8)	−1.4 (−34 to 0.7)	0.512
HAQ at baseline	1.15 (0.25–2.88)	1.25 (0–2.88)	0.869
HAQ at 6 months	0.75 (0–2.13)	0.57 (0–2.88)	0.633
HAQ at 12 months	0.75 (0–1.38)	0.63 (0–2.75)	0.826
**Δ**_1_HAQ	−0.25 (−1.87 to 0.98)	−0.38 (−2.75 to 1.5)	0.674
**Δ**_2_HAQ	−0.31 (−1.25 to 0)	−0.19 (−2.75 to 0.38)	0.338
Patient pain VAS at baseline	75 (40–100)	52 (0–100)	0.003
Patient pain VAS at 6 months	48 (8–80)	30.5 (0–100)	0.353
Patient pain VAS at 12 months	45 (0–80)	39 (0–80)	0.486
Patient pain **Δ**_1_VAS	−30 (−59 to 2)	−30 (−85 to 11)	0.431
Patient pain **Δ**_2_VAS	−27.5 (−75 to 30)	−33 (−70 to 50)	0.838
Patient fatigue VAS at baseline	68 (0–90)	50 (0–100)	0.006
Patient fatigue VAS at 6 months	50 (4–71)	40.5 (0–100)	0.285
Patient fatigue VAS at 12 months	40 (10–79)	36.5 (0–100)	0.739
Patient fatigue **Δ**_1_VAS	−20 (−58 to 1)	−20 (−95 to 28)	0.971
Patient fatigue **Δ**_2_VAS	−32.5 (−75 to 16)	−20 (−60 to 43)	0.462
Patient global VAS at baseline	72 (21–100)	50 (0–100)	0.006
Patient global VAS at 6 months	47 (5–70)	49.5 (0–86)	0.791
Patient global VAS at 12 months	50 (0–80)	30 (0–82)	0.374
Patient global **Δ**_1_VAS	−20 (−55–0)	−30 (−86 to 40)	0.51
Patient global **Δ**_2_VAS	−20 (−75 to 30)	−30 (−80 to 40)	0.984
Physician global VAS at baseline	61.5 (6–90)	50 (0–90)	0.121
Physician global VAS at 6 months	27.5 (10–54)	20 (0–63)	0.092
Physician global VAS at 12 months	20 (0–70)	13 (0–68)	0.612
Physician global **Δ**_1_VAS	−43 (−63 to 4)	−32.5 (−80 to 20)	0.625
Physician global **Δ**_2_VAS	−47.5 (−85 to 0)	−40 (−90 to 28)	0.355

Data are expressed as the median (min–max). Δ_1_ reflects the changes between baseline and 6 months. Δ_2_ reflects the changes between baseline and 12 months.
